# Expression profiling of the retina of pde6c, a zebrafish model of retinal degeneration

**DOI:** 10.1038/sdata.2017.182

**Published:** 2017-12-12

**Authors:** Liyun Zhang, Xinlian Zhang, Gaonan Zhang, Chi Pui Pang, Yuk Fai Leung, Mingzhi Zhang, Wenxuan Zhong

**Affiliations:** 1 Department of Biological Sciences, Purdue University, 915 W. State Street, West Lafayette, IN 47907, USA; 2 Department of Statistics, University of Georgia, 101 Cedar St, Athens, GA 30602, USA; 3 Department of Ophthalmology and Visual Sciences, Chinese University of Hong Kong, Hong Kong; 4 Department of Biochemistry and Molecular Biology, Indiana University School of Medicine, Indianapolis, IN 46202, USA; 5 Purdue Institute for Drug Discovery, 610 Purdue Mall, Purdue University, West Lafayette, IN 47907, USA; 6 Purdue Institute for Integrative Neuroscience, Purdue University, 610 Purdue Mall, West Lafayette, IN 47907, USA; 7 Joint Shantou International Eye Center, Shantou University & the Chinese University of Hong Kong, Shantou 515041, China

**Keywords:** RNA sequencing, Gene expression, Zebrafish, Retinal diseases

## Abstract

Retinal degeneration often affects the whole retina even though the disease-causing gene is specifically expressed in the light-sensitive photoreceptors. The molecular basis of the retinal defect can potentially be determined by gene-expression profiling of the whole retina. In this study, we measured the gene-expression profile of retinas microdissected from a zebrafish *pde6c*^*w59*^ (*pde6c*) mutant. This retinal-degeneration model not only displays cone degeneration caused by a cone-specific mutation, but also other secondary cellular changes starting from 4 days postfertilization (dpf). To capture the underlying molecular changes, we subjected *pde6c* and wild-type (WT) retinas at 5 dpf/ 120 h postfertilization (hpf) to RNA sequencing (RNA-Seq) on the Illumina HiSeq 2,000 platform. We also validated the RNA-Seq results by Reverse Transcription Quantitative Polymerase Chain Reaction (RT-qPCR) of seven phototransduction genes. Our analyses indicate that the RNA-Seq dataset was of high quality, and effectively captured the molecular changes in the whole *pde6c* retina. This dataset will facilitate the characterization of the molecular defects in the *pde6c* retina at the initial stage of retinal degeneration.

## Background & Summary

In retinal degenerative diseases, light-sensitive photoreceptors often die due to mutations in genes specifically expressed in these cells. This photoreceptor-specific defect will lead to a series of cellular changes, which would ultimately affect the well-being of the whole retina. For example, in a zebrafish mutant *pde6c*^*w59*^ (*pde6c*), an A>G point mutation was identified in the *pde6c (phosphodiesterase 6C, cGMP-specific, cone, alpha prime)* gene^1^. This mutation was predicted to cause a frameshift in the coding sequence and result either in a truncated PDE6C or degradation of *pde6c* mRNA through nonsense-mediated decay. This mutation ultimately affects both cone and rod photoreceptors.

In wild-type zebrafish retina, photoreceptor progenitors withdraw from the cell cycle at 48 h post-fertilization (hpf) to form the photoreceptor precursors in the outer nuclear layer (ONL)^2^. These precursors will begin to differentiate into rods and cones at 50 hpf in a ventral part of the retina^3^. Then, cones gradually distribute evenly throughout retina; whereas rods distribute sporadically in the retina, with higher density in the ventral region. By 3 dpf, these photoreceptors are mature enough that the fish larvae will display the first visually-evoked startle response^4^. In *pde6c*, cones seem to develop following the normal course. They form outer segment by 3 dpf, but they degenerate starting at 4 dpf. *Pde6c* rods die subsequently as bystanders^1^, even though they do not express the *pde6c* gene. They also develop first in the retina, but they appear abnormal with more pronounced outer segment at 4 dpf^1^, when cones first degenerate. Nonetheless, rod number is comparable to WT at least up to 6 dpf^5^, and only begins to reduce in the central retina by 8 dpf^1^. The degeneration of these photoreceptors will also cause reactive gliosis as early as 7 dpf^6^, and abnormal morphology in bipolar cells with displaced nuclei and axonal processes at 8 dpf^1^. These tissue-level consequences are all initiated by a cone-specific *pde6c* mutation. The molecular basis of these retinal defects can often be analysed by profiling gene expression of the whole retina^7–10^, which can detect expression change even in a few cells^11^. Such expression profiles will facilitate the study of gene-regulatory mechanisms for these pathological events.

In this study, we measured the gene expression in the *pde6c* mutant retina and WT retina by RNA sequencing (RNA-Seq). We conducted RNA-Seq at 5 dpf/ 120 hpf. At this stage, cones have substantially degenerated, whereas the bystander rods display abnormal morphology^1,5^. The Müller cells also display phenotype that is consistent with reactive gliosis (unpublished observation). Therefore, measuring retinal gene expression at 5 dpf/ 120 hpf would give us a glimpse of the global retinal defects at the initial stage of retinal degeneration. We analysed the sequencing results by various exploratory analyses, which indicate our RNA-Seq dataset was of high quality. We also evaluated the quality of our RNA-Seq results by reverse-transcription quantitative polymerase chain reaction (RT-qPCR), using a few key phototransduction genes. We observed a high correlation of fold changes of these genes as measured by both techniques. This observation strongly implicates that our RNA-Seq dataset effectively measured the expression changes in the *pde6c* retina. This dataset will therefore facilitate downstream characterization of the molecular problems in the *pde6c* retina.

## Methods

### Fish maintenance and embryo collection

The *pde6c*^*w59*^ (*pde6c*) mutant line^1^ was purchased from the Zebrafish International Resource Center (ZIRC; http://zebrafish.org/) and maintained according to standard procedures^12^. The embryos were collected at 15-min intervals and raised at 28 °C in E3 medium^13^. The breeding of parents was also staggered so that the embryonic retinas could all be dissected and collected at 120 hpf. The homozygous mutant embryos (i.e., *pde6c*^*w59/w59*^) were obtained from the cross of the *pde6c* heterozygous parents, whereas the WT-control embryos were collected from the cross of genotyped WT siblings of the *pde6c* heterozygous parents. All protocols were approved by the Purdue Animal Care and Use Committee.

### Optokinetic response (OKR)

An OKR apparatus was constructed based on the specifications as described previously^14^. This machine was used to measure the OKR of the zebrafish larvae. During the assay, the larvae were partially immobilized in 3% methylcellulose in a 35-mm Petri dish. The dish was placed in the center of a circular drum with 20° black and white vertical stripes attached on the inner surface. These stripes were illuminated by a Fiber Lite M1–150 illuminator (Dolan-Jenner Industries, Boxborough, MA). The illuminance was approximately 20,000 Lux at the level of the Petri dish, as measured by a LX1010B light meter (Mastech, Taipei, Taiwan). During the OKR measurement, the rotation speed of the drum was set at 8 revolutions per minute. In response to stripe rotation, normal larval eyes would display a characteristic smooth pursuit of rotation, and a rapid, corrective movement called saccade.

### Genotyping

The *pde6c*^*w59*^ mutation was genotyped as previously described^5^. In short, DNA was extracted from adult tail fin or larval body after retinal dissection. Then, the extracted DNA was used to amplify a 157-bps fragment from the *pde6c* gene with a specific pair of primers (Table 1). During amplification, a restriction site BsaXI was created only in the mutated *pde6c*^*w59*^ allele but not in the WT allele. This difference was discriminated by restriction analysis of these DNA fragments by BsaXI. On the resulting agarose gel, a WT allele would give a 157-bps band, whereas the *pde6c*^*w59*^ allele would give a 122-bps and a 35-bps band.

### Micro-dissection of zebrafish retina

WT retinas and *pde6c*^*w59/w59*^ retinas were microdissected from 5-dpf larvae using a previously published protocol^7,15^. These larvae were first screened by the OKR assay. This non-invasive screening streamlined the identification of *pde6c*^*w59/w59*^ larvae from the cross of heterozygous parents, as only ¼ of the clutch would be homozygotes and they looked phenotypically normal. All larvae were dark-adapted for at least 2 h before dissection. Then, three retinas were dissected from three independent larvae and combined as one biological replicate. The remaining larval trunk was further genotyped to confirm its genetic identity. Three biological replicates were finally collected for each genotype (Table 2).

### Total RNA extraction, characterization and amplification

Total RNAs was extracted from the biological replicates by an optimized procedure^7,8^ that combined TRIzol (Life Technologies, Grand Island, NY) and RNeasy Micro kit (Qiagen, Valencia, CA). The quality of the extracted total RNAs were evaluated by Bioanalyzer electrophoresis (Agilent Technologies, Santa Clara, CA). The RIN values were 9.1, 9.1 and 8.9 for the three replicates of WT retinas, and were 9.5, 9.1 and 9.3 for the three replicates of *pde6c* retinas (Table 2). Then, one nanogram of the total RNA from each group was amplified by Ovation RNA-Seq System V2 (NuGEN Technologies, San Carlos, CA). The yield of the amplified cDNA products was evaluated by Nanodrop spectrophotometry (Thermo Scientific).

### RNA sequencing and analysis

For each replicate, one microgram of the amplified cDNA was paired-end sequenced by Illumina HiSeq 2,000 (Illumina, Inc., San Diego, CA). The six samples—three WT retinas (WR1, WR2, and WR3 in Table 2) and three *pde6c* retinas (PR1, PR2, and PR3 in Table 2)—were combined and run on three different lanes. Running all samples in one lane eliminated lane-to-lane variation^16^, whereas running the samples on multiple lanes increased coverage. It should be noted that another six unrelated samples were included in these three lanes during RNA sequencing. This reduced the theoretical coverage of each sample by half.

### Sequencing quality check and read alignment

**Figure 1:**
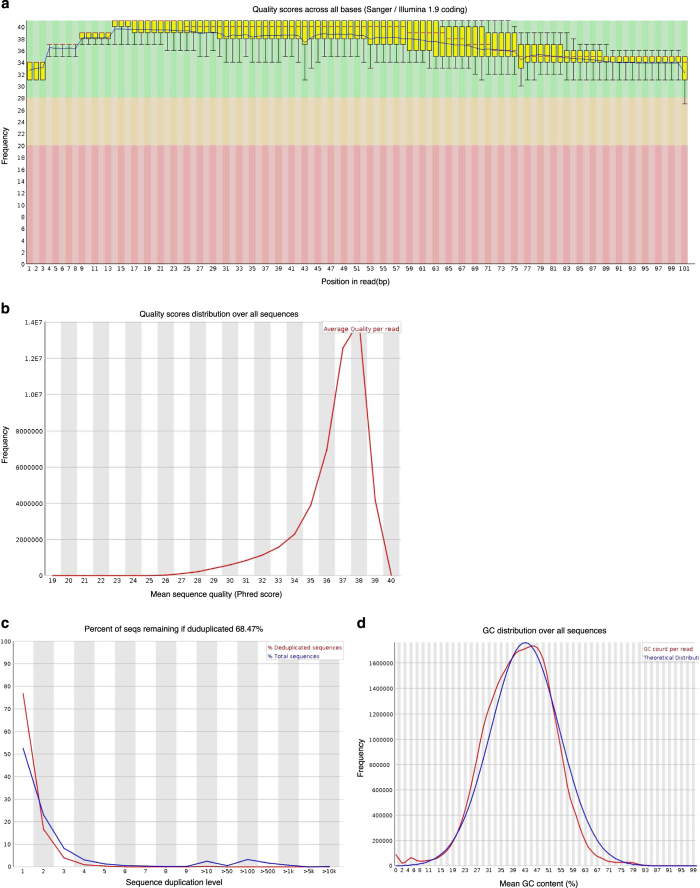
Quality assessment of raw FASTQ sequence data for paired-end reads of one sample (read 1 of sample PR1). (**a**) Box plots of the distribution of per-base quality scores; (**b**) Distribution of quality scores of all sequences; (**c**) Percentage of sequences with different degrees of duplication in both total sequences (blue line) and de-deduplicated sequences (red line). (**d**) The distribution of GC content (%) over all sequences compared to the theoretical normal distribution. All figures were generated using the FastQC v0.11.5 program.

The raw fastq reads were trimmed for adapters and preprocessed to remove low-quality reads using Trimmomatic version 0.3^17^ with default parameter setting for Illumina paired-end reads. The reads were aligned to primers and/or adaptors with no more than two mismatches. Reads shorter than 36 bases were removed. After adapter removal, the quality of each paired-end sequence file was evaluated using FastQC analysis http://www.bioinformatics.babraham.ac.uk/projects/fastqc/ (see Code Availability 1) based on the following parameters: 1) Distribution of quality score (Phred score) per base, 2) Distribution of quality scores of the raw sequences, 3) Distribution of duplicated reads, and 4) GC content (%) distribution of the raw sequences. The analyses indicate that our sequencing was of high quality, as illustrated by a typical sample in Fig. 1. First, we evaluated quality of sequences by plotting the boxplots for Phred scores per base (Fig. 1a) and distribution of average Phred scores of all sequences (Fig. 1b). Both the Phred score per base and average Phred score of all sequences are generally above 28, the default threshold for a high-quality base call. Second, we confirmed the diversity of library sequences by plotting a duplication plot (Fig. 1c), which shows the proportion of library sequences with different levels of duplication. The blue line shows the full sequence set and how its duplication levels are distributed. The red line shows the sequences after removal of duplicated reads, i.e., de-duplicated set. In both cases, most sequences fall into the far left of the plot, indicating that the library was diverse and most sequences occurred only a small number of times in the final set. The blue lines are relatively flat compared to red line, thus do not indicate significant contamination or any severe technical duplication. The duplicated reads constitute about 30% of all reads (Fig. 1c). Third, we compared the distribution of sequence GC content (%) to the theoretical normal distribution (Fig. 1d). The two curves substantially overlap with each other, indicating that the adaptors removal was successful and there is no obvious contamination.

These reads were then aligned to the zebrafish genome, release 89 <ftp://ftp.ensembl.org/pub/release-89/> using STAR v2.5.3a^18^ (see Code Availability 1). The aligned reads were then sorted and indexed using SAMTools v1.4.1^19^. The key outputs of alignment are shown in Table 3. These include the number of input reads, average input read length, uniquely mapped read, and mismatch rate per base. The uniquely mapped reads rate is around 70% and the mismatch rate per base is only about 1%. These observations indicate that the quality of all sequencing results was satisfactory.

### Read counts, normalization, and differential gene expression

**Figure 2:**
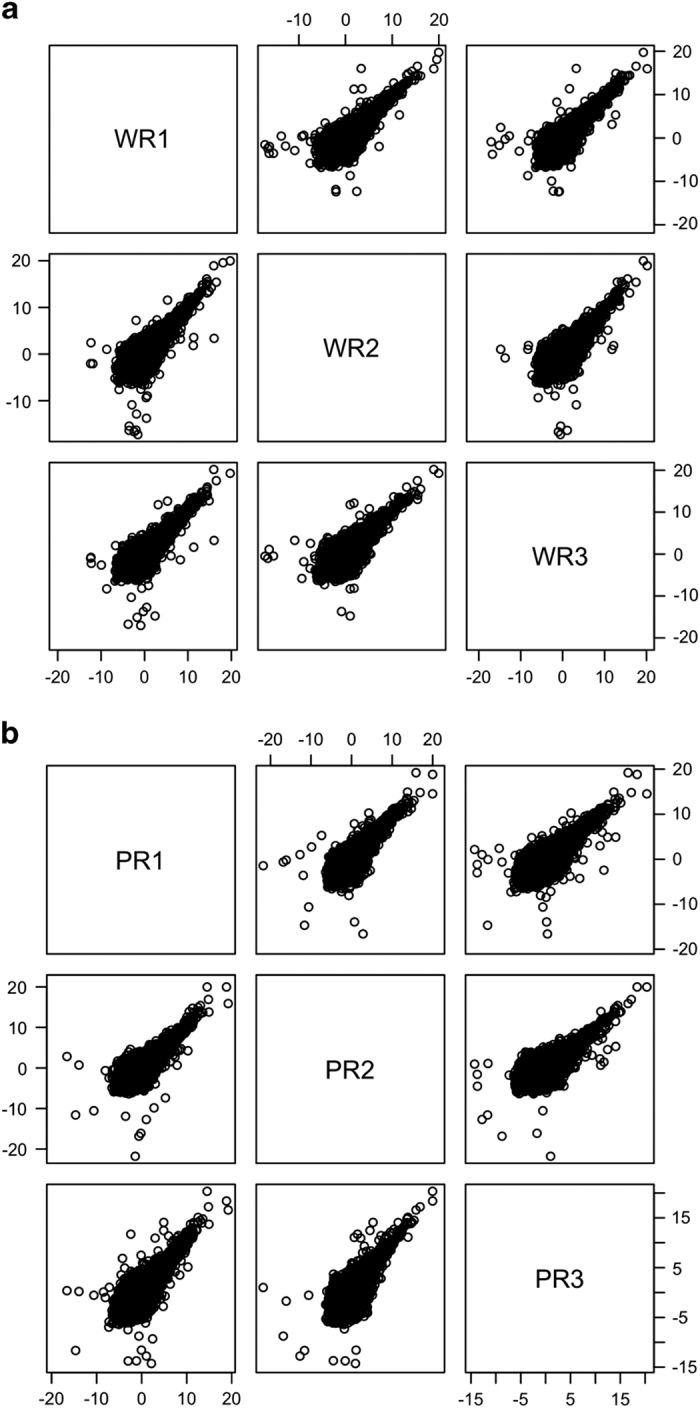
Scatter plots of log2-transformed FPKM: (**a**) Pairwise scatter plots of the log2-transformed FPKM (output from Cufflinks v2.2.1) of all genes in the 3 WT samples (WR1, WR2, WR3); (**b**) Pairwise scatter plots of the log2-transformed FPKM (output from Cufflinks v2.2.1) of all genes in the 3 *pde6c* samples (PR1, PR2, PR3).

To determine transcript abundance, we used the aligned reads to calculate the Fragments Per Kilobase of transcript per Million mapped reads (FPKM) by Cufflinks v2.2.1^20^(see Code Availability 1). The percentage of genes with nonzero FPKM in all samples are around 65% and shown in Table 4. We compared the FPKMs of the three biological replicates of the same genotype in pairwise scatterplots (Fig. 2). Most values on the scatterplots fall along the y=x line, supporting the consistency among the biological replicates.

**Figure 3:**
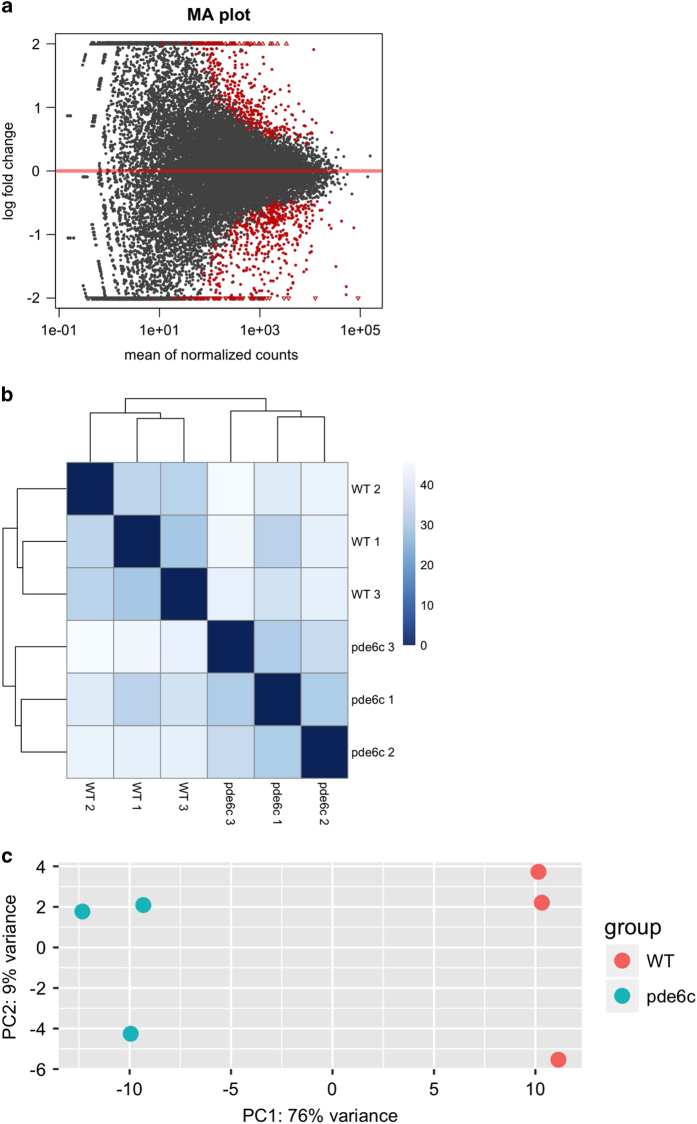
Exploratory analyses of the RNA-seq data. (**a**) MA plot of the log2 fold change of all genes. Red points indicate genes with *P* value less than 0.1 and points; whereas red triangles indicate genes with log2 fold change greater than 2 or less than −2. (**b**) Heatmap of 3 wild type samples (WR1, WR2, WR3) and 3 *pde6c* samples (PR1, PR2, PR3) using the Euclidean distance of the log2-transformed counts (after library size normalized and variance stabilized). A darker colour means a smaller Euclidean distance, i.e., more correlated. (**c**) PCA plot using the log2-transformed counts (after library size normalization and variance stabilization). These plots were generated based on the output from DESeq 2 v1.16.1.

We also summarized read counts aligned to protein-coding genes in the gene transfer format (gtf) file from Ensemble Genome release 89 by featureCounts v1.5.2^21^ (see Code Availability 1). The output of featureCounts was fed to the package DESeq2 v1.16.1^22^ in R statistical environment (see Code Availability 1) to identify differentially expressed genes. This package normalized the count data against library size, log2-transformed the normalized data, calculated fold change between samples, inferred significance using a model with negative binomial distribution, and adjusted for multiple hypothesis testing. The resulting log2 fold changes of all samples were then plotted in an MA plot (Fig. 3a), which shows the fold-change values of genes against the mean of normalized counts of all samples. These fold changes show a typical pattern for genome-wide experiments: the majority of the genes did not show a significant change in expression (log2 fold change about 0); whereas only a few genes showed a significant differential expression (Wald significance test^22^; adjusted *P*-value less than 0.1). These differentially-expressed genes are highlighted as red points in the plot. If the genes have a log2 fold change either greater than 2 or less than −2, they are plotted as open triangles.

Then, we further validate the reproducibility across the biological replicates by hierarchical clustering (HC) (Fig. 3b) and principle component analysis (PCA) (Fig. 3c), using the replicates’ log2-transformed counts after library-size normalization and variance stabilization (see Code Availability 1). In the HC heatmap (Fig. 3b), the 3 WT samples (WR1, WR2, WR3) are clustered together and the 3 *pde6c* samples (PR1, PR2, PR3) are clustered together. In the PCA plot (Fig. 3c), these WT and *pde6c* samples are well separated from each other by their difference in the first PC, which explained 76% of the variance in the expression variables. Together, these sequencing analyses suggest that our RNA-seq had consistently measured the WR and PR samples, and effectively captured their biological difference.

### Code availability

1. Code used for quality assessment and data analysis in this study is available at: https://gist.github.com/coralzhang/fc4e51609ff316486c1682feed6404a9/471afb15b7f7b230a38e4eedaadcf5f679412a07.

## Data Records

Raw FASTQ files for the RNA-seq libraries were deposited to the NCBI *Gene Expression Omnibus (GEO)* with accession number GSE101544 and have been assigned BioProject accession PRJNA394760 (Data Citation 1) and SRA number SRP112616 (Data Citation 2). Key outputs of the analysis were deposited to the *GEO* with the same accession number (Data Citation 1) providing access to all relevant data files.

## Technical Validation

To validate the RNA-Seq results, we collected independent samples and analyzed the expression difference of selected genes by RT-qPCR. First, 30 eyes were microdissected from 15 larvae in both WT and *pde6c* groups. Then, total RNAs were extracted from these eye samples and reverse-transcribed into cDNAs as described^7^. These cDNAs were used for RT-qPCR reactions using SYBR Green PCR Master Mix (Life Technologies, Grand Island, NY). The RT-qPCR primers were designed with the RealTime PCR program (Integrated DNA Technologies, Coralville, IA) (Table 1). These RT-qPCR reactions were run on an Applied Biosystems 7,300 Real-Time PCR System (Life Technologies, Grand Island, NY) according to manufacturer’s instructions.

**Figure 4:**
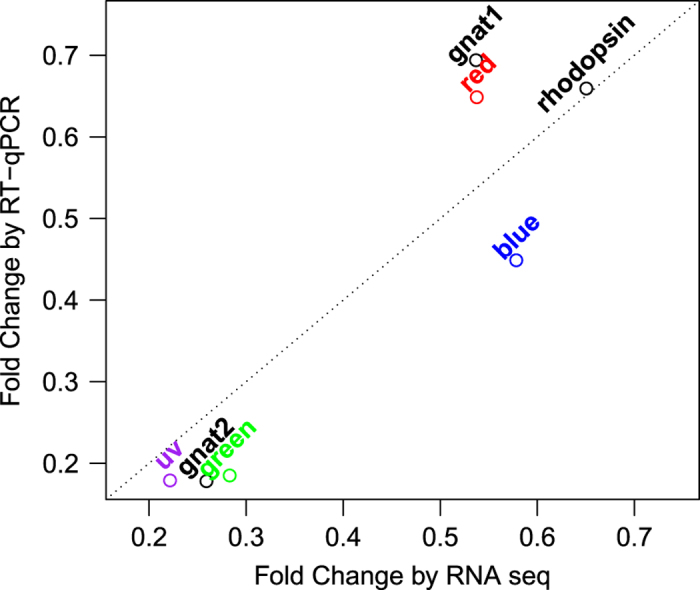
Validation of RNA-Seq data by RT-qPCR analysis. The fold changes by RNA-Seq were based on the output from DESeq 2 v1.16.1. The plot shows *rhodopsin* (*rho*), four cone *opsins* (*red, green, blue,* and *uv),* and two *transducins* (*gnat1* and *gnat2*).

In the RT-qPCR analysis, we measured the expression of seven phototransduction genes: *rhodopsin* (*rho*), four cone *opsins* (*red, green, blue,* and *uv)*, and two *transducins* (*gnat1* and *gnat2*). β-actin was used as the normalization control. We collected gene expressions in duplicates from independently-dissected eyes, and calculated a fold change between *pde6c* and WT samples for each duplicate using the ΔΔCt method^23^. Then, we averaged the fold changes of the duplicates. The resulting fold changes highly correlated to those fold changes obtained by RNA-Seq (Fig. 4; r=0.91, *t*-test *P* value=0.0039). This high correlation strongly suggests that the RNA-Seq data effectively captured the gene-expression change of the *pde6c* retina. This RNA-Seq dataset will likely facilitate the characterization of the molecular defects in different cell types of the *pde6c* retina.

## Additional information

**How to cite this article:** Zhang, L. *et al.* Expression profiling of the retina of pde6c, a zebrafish model of retinal degeneration. *Sci. Data* 4:170182 doi: 10.1038/sdata.2017.182 (2017).

**Publisher’s note:** Springer Nature remains neutral with regard to jurisdictional claims in published maps and institutional affiliations.

## Supplementary Material



## Figures and Tables

**Table 1:** Primers used for genotyping and validation of the RNA-Seq dataset.

**Gene**	**Forward Primer**	**Reverse Primer**	**Remark**
*pde6c*	TTGGCCTCTGGAATACTGGCTCTC	GTTTGACCAGAACCCGGAAG	For genotyping
*gnat1*	CGTCAAGTTTGTGTTCGATGC	GAGGAAACGAGCTACAAGGAG	For RT-qPCR
*gnat2*	CAAACCTGACTACCTTCCCAC	TCTTCCTCTCGGACCTCTG	For RT-qPCR
*opn1lw2 (red opsin)*	CCAACAGCAATAACACAAGGG	GCGACAACCACAAAGAACATC	For RT-qPCR
*opn1mw1 (green opsin)*	GGCTGTGTAATGGAGGGATTC	ATGGTTTGCGGAGAATTTGAAG	For RT-qPCR
*opn1sw2 (blue opsin)*	GGTTCCTTTCAGCACCATTG	AGAAGCCGAACACCATTACC	For RT-qPCR
*opn1sw1 (uv opsin)*	TCATTTTCTCCTACTCACAGCTC	CACAAAAGAGCCAACCATCAC	For RT-qPCR
*rhodopsin*	AGTCCTGCCCAGACATCTAG	GTACTGTGGGTATTCGTATGGG	For RT-qPCR
*β-actin*	TGCTGTTTTCCCCTCCATTG	GTCCCATGCCAACCATCACT	For RT-qPCR

**Table 2:** Samples used in this study.

**Source**	**Protocol 1**	**Protocol 2**	**Samples**	**Protocol 3**	**Protocol 4**	**Data Citation**
Wild-type retina	Retinal dissection	TRIzol+RNeasy Micro	WR1 (Biological Replicate 1)	Ovation RNA-Seq System V2	Illumina HiSeq 2000	Data Citation 1 GSM2705944
Wild-type retina	Retinal dissection	TRIzol+RNeasy Micro	WR2 (Biological Replicate 2)	Ovation RNA-Seq System V2	Illumina HiSeq 2000	Data Citation 1 GSM2705945
Wild-type retina	Retinal dissection	TRIzol+RNeasy Micro	WR3 (Biological Replicate 3)	Ovation RNA-Seq System V2	Illumina HiSeq 2000	Data Citation 1 GSM2705946
*pde6c* retina	Retinal dissection	TRIzol+RNeasy Micro	PR1 (Biological Replicate 1}	Ovation RNA-Seq System V2	Illumina HiSeq 2000	Data Citation 1 GSM2705947
*pde6c* retina	Retinal dissection	TRIzol+RNeasy Micro	PR2 (Biological Replicate 2)	Ovation RNA-Seq System V2	Illumina HiSeq 2000	Data Citation 1 GSM2705948
*pde6c* retina	Retinal dissection	TRIzol+RNeasy Micro	PR3 (Biological Replicate 3)	Ovation RNA-Seq System V2	Illumina HiSeq 2000	Data Citation 1 GSM2705949

**Table 3:** Details of key QC metrics of RNA-seq library after alignment with STAR v2.5.3a.

**Sample**	**WR1**	**WR2**	**WR3**	**PR1**	**PR2**	**PR3**
Number of input reads	48960995	46550508	47008978	51108456	42986393	49621925
Ave. input read length	197	197	197	197	197	196
Uniquely mapped reads	70.16%	71.35%	70.14%	69.34%	70.07%	71.04%
Mismatch rate per base	1.42%	1.45%	1.54%	1.48%	1.57%	1.52%
*Note that STAR counts a paired-end read as one read and that the default for max number of mismatches for a pair end sequence (2×100b) is 8 per pair^18^.						

**Table 4:** Percentage of genes with nonzero FPKM (output from Cufflinks v2.2.1).

**Sample**	**WR1**	**WR2**	**WR3**	**PR1**	**PR2**	**PR3**
Percentage	65.85%	61.21%	61.29%	65.25%	60.33%	63.03%
